# Retraction: Lyons-Weiler, J.; Thomas, P.Incidence of Office Visits and Cumulative Rates of Billed Diagnoses along the Axis of Vaccination. *Int. J. Environ. Res. Public Health* 2020, *17*, 8674

**DOI:** 10.3390/ijerph18157754

**Published:** 2021-07-22

**Authors:** IJERPH Editorial Office

**Affiliations:** MDPI, St. Alban-Anlage 66, 4052 Basel, Switzerland; ijerph@mdpi.com

The journal retracts the article “Relative Incidence of Office Visits and Cumulative Rates of Billed Diagnoses along the Axis of Vaccination” cited above [1]. Following publication, concerns were brought to the attention of the editorial office regarding the validity of the conclusions of the published research. 

Adhering to our complaints procedure, an investigation was conducted that raised several methodological issues and confirmed that the conclusions were not supported by strong scientific data. The article is therefore retracted. 

This retraction is approved by the Editor in Chief of the journal.

The authors did not agree to this retraction.
